# Quantitative Analysis of Postural Balance in Faller and Nonfaller Patients with Parkinson's Disease

**DOI:** 10.1155/2023/9688025

**Published:** 2023-06-20

**Authors:** Do Young Kwon, Yuri Kwon, Ji-An Choi, Junghyuk Ko, Ji-Won Kim

**Affiliations:** ^1^Department of Neurology, Korea University College of Medicine, Ansan-si 15355, Republic of Korea; ^2^Department of Biomedical Engineering, Konkuk University, Chungju-si, Chungcheongbuk-do 27478, Republic of Korea; ^3^Research Institute of Biomedical Engineering, Konkuk University, Chungju-si, Chungcheongbuk-do 27478, Republic of Korea; ^4^Division of Mechanical Engineering, College of Engineering, Korea Maritime and Ocean University, Busan 49112, Republic of Korea

## Abstract

**Background:**

Postural instability has been identified as a fall risk factor with a significant impact on the quality of life of patients with Parkinson's disease (PD). The aim of this study was to compare the center of pressure (COP) between faller and nonfaller patients with PD during static standing.

**Methods:**

Thirty-two faller patients and 32 nonfaller patients with PD participated in this study. All patients performed the static balance test on a force plate. COP data were recorded during quiet standing. Mean distance, sway area, mean velocity, mean frequency, and peak power were derived from the COP data. Statistical analysis was performed using independent *t*-tests to compare faller and nonfaller patients.

**Results:**

Fallers presented a greater average distance, wider sway area, faster average speed, and greater peak power than nonfallers (*p* < 0.05). In contrast, no significant group differences were observed in peak frequency and mean frequency (*p* > 0.05).

**Conclusions:**

Although falls occur during dynamic activities, our study demonstrated that even a safe and simple static postural balance test could significantly differentiate between faller and nonfaller patients. Thus, these results suggest that quantitatively assessed static postural sway variables would be useful for distinguishing prospective fallers among PD patients.

## 1. Introduction

Postural instability is one of the representative clinical characteristics in Parkinson's disease (PD) and occurs due to deteriorated neuromuscular functions such as postural deformities, the loss of postural adaptation reflexes, and abnormal central processing. Postural instability increases the risk of falls in patients with PD [1, 2]. Indeed, 50–70% of the patients with PD experience falls [3, 4], which is approximately twice that of normal elderly adults [4]. Furthermore, patients with PD are also nine times more likely to experience recurrent falls compared to normal elderly adults [1]. Falls represent the third-highest cause of hospitalization [5]. Therefore, it is important to quantitatively evaluate postural balance because an accurate assessment could be useful for fall prevention in patients with PD [6]. In addition, understanding the postural balance strategy in PD might be helpful for identifying fall risk factors and determining appropriate nonpharmacological interventions to improve the patient's quality of life.

Generally, a force plate provides a safe, convenient, quantitative, and sensitive measurement of postural instability in PD patients. Therefore, some studies measured static postural balance and suggested that PD patients showed greater lateral sway compared to healthy elderly adults [6]. Additionally, greater sway areas, as well as longer sway paths, were observed, even in the early stages of PD [7]. However, these studies focused only on the comparisons of PD patients and healthy elderly adults. It is important to characterize postural balance in patients with fall histories to predict the fall risk among PD patients. A better understanding of the differences between fallers and nonfallers may provide further insight into fall risk assessment in this clinical population.

The aim of this study was to quantitatively compare postural balance between faller and nonfaller patients with PD during static standing. We hypothesized that some COP-based outcome measures could differentiate faller from nonfaller patients with PD.

## 2. Materials and Methods

Sixty-four PD patients (32 faller patients and 32 nonfaller patients) participated in a balance test. All patients were diagnosed by the United Kingdom Brain Bank criteria (UKBBC) and were recruited from a university-affiliated hospital. PD patients were divided into fallers and nonfallers based on the self-reported occurrence of falls in the previous 1-year (12 months). Specifically, the fallers were defined as patients that had a history of at least one fall recorded over a 1-year period by personal interview. Of the faller patients, 16 had fallen fewer than 10 times and 16 had fallen more than 10 times. Each patient group had the same gender ratio (14 men and 18 women). Two neurologists administered unified Parkinson's disease rating scale (UPDRS) part III assessments, the new freezing of gait questionnaire (NFOGQ), fear of falling (FOF) assessments, the Tinetti balance test, frontal assessment battery (K-FAB), patient health questionnaire-9 (PHQ-9), and the state-trait anxiety inventory (STAI) to all patients. Patients who could walk and stand independently without assistive devices were included in our study. Additionally, the patients included in this study had the following: age of <85 years, disease duration of ≤15 years, and Hoehn and Yahr stage ≤3. Moreover, the patients who have no history of diseases that might affect the function of balance and gait such as diabetes, vestibular disorders, and severe musculoskeletal problems were recruited. PD patients with a familial history of parkinsonism, a previous history of stroke, peripheral neuropathy of any cause, psychiatric disorders, possible signs of a typical parkinsonism, and those taking medications that affect central dopaminergic pathways were excluded from the study. Patients with PD dementia according to the Movement Disorder Society PD criteria were also excluded. All patients provided informed consent, and the study was approved by the KOREA university hospital Ethics Committee.

A custom-made force platform (327 mm × 312 mm) validated in a previous study [8–10] was used to measure the anteroposterior (AP) and medio–lateral (ML) COP data. The force platform incorporated a force sensor in each of the four corners of the platform and the COP data were sent to a PC tablet via serial communication. LabVIEW software was used to develop the monitoring and data recording system for the COP data in the ML and AP directions.

All patients performed the balance test on the force platform while in a quiet upright standing position. The balance test was performed in the optimally medicated state (dopaminergic medications). Three 30-s trials were separated by a rest break to avoid fatigue. The footprints were drawn on a paper affixed to the force platform in order to minimize variability and maintain same feet position between repeated balance tests. All patients were asked to stand as stably as possible at a preferred, comfortable feet-apart-width stance and to stare at a 10-cm black colored circle fixation mark on a wall. AP and ML COP data were filtered using a low-pass filter [11, 12]. COP outcome measures including mean velocity, mean distance, mean frequency, peak frequency, peak power, and sway area were derived from the filtered COP data. MATLAB software (version R2020b, MathWorks, Inc., Natick, MA, USA) was used to calculate the COP outcome measures. The average of the three trials was used for subsequent analysis. An independent *t*-test was used to compare COP outcome measures between the faller and nonfaller groups using SPSS software. In addition, Spearman correlation analysis was performed to identify the relationship between COP outcome measures and clinical scores.

## 3. Results and Discussion

### 3.1. Results

Table 1 shows subject information and the clinical scores in the faller and nonfaller patients. There were no significant differences in age, height, weight, or education periods between faller and nonfaller patients. Disease duration and the Hoehn and Yahr stages of the fallers were significantly longer and higher, respectively, than those of nonfallers (*p* < 0.05), whereas there was no significant difference in UPDRS part III (*p* > 0.05). In clinical assessments, NFOGQ, K-FAB, and STAI scores were not statistically different between the groups (*p* > 0.05), whereas there were significant differences in FOF and Tinetti balance scores between the groups (*p* < 0.05).

**Figure 1:**
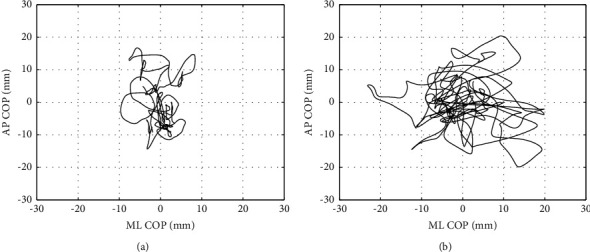
Representative COP trajectory of nonfaller (a) and faller (b) patients.

Figure 1 shows representative COP distance trajectories in nonfaller and faller patients. Faller patients showed increased magnitude, particularly in the ML direction compared with nonfaller patients.

**Figure 2:**
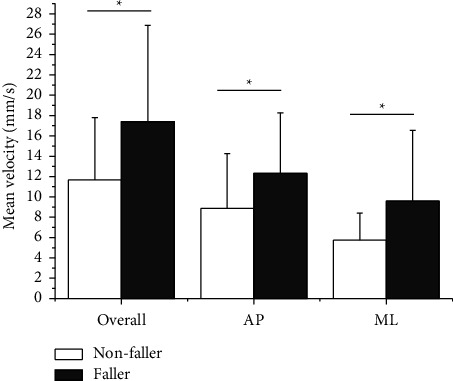
Mean velocity in nonfaller patients and faller patients. (^*∗*^*p* < 0.05).

**Figure 3:**
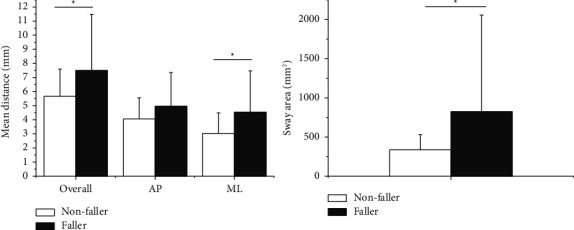
Mean distance and sway area in nonfaller patients and faller patients. (^*∗*^*p* < 0.05).

**Figure 4:**
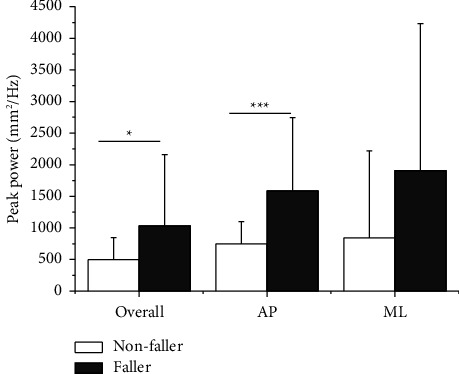
Peak power in nonfaller patients and faller patients. (^*∗*^*p* < 0.05, ^*∗∗∗*^*p* < 0.001).

Table 2 shows a comparison of COP outcome measures between faller and nonfaller patients. In both the AP and ML directions, the mean velocity was significantly faster in faller than in nonfaller patients during static standing as shown in Figure 2 (*p* < 0.05).  Figure 3 shows the results of the mean distance and sway area. The mean distance was significantly greater in faller than in nonfaller patients, particularly in the ML direction (*p* < 0.05). In addition, the sway area was significantly wider in faller patients compared to nonfaller patients (*p* < 0.05). As shown in Figure 4, faller patients showed greater peak power, particularly in the AP direction compared to nonfaller patients (*p* < 0.05). In contrast, no significant differences were found between the groups in mean frequency and peak frequency.

Table 3 shows the results of correlation analysis between COP outcome measure and clinical scores. Tinetti balance score was significantly correlated with mean velocity, mean distance, peak power, and sway area (*r* = −0.366∼−0.464, *p* < 0.01). Fear of falling score showed weaker correlation compared to Tinetti balance score (*r* = 0.264∼0.383, *p* < 0.05). No significant correlation was observed in UPDRS III total score (*p* > 0.05).

### 3.2. Discussion

In this study, static balance performance was quantitatively investigated in faller patients and compared to nonfaller patients with PD. The main findings of this study were as follows. First, faller patients exhibited significantly different postural sway sizes (greater ML sway and wider area) than nonfaller patients, even though they did not complain of instability during repeated examinations. Second, faller patients showed faster postural sway speed than nonfaller patients. Third, the main power component of AP postural sway was greater in faller patients than in nonfaller patients.

The HY stage of faller patients was significantly greater than that of nonfaller patients (*p* < 0.05) even though there was no significant difference in UPDRS III total scores (*p* > 0.05). This indicates that the HY stage is more associated with balance ability rather than the UPDRS III total score. Indeed, the UPDRS III total score includes various motion functions, such as tremor and bradykinesia, as well as postural instability. Postural balance was affected in PD patients with FOG more significantly than that in non-FOG patients [13]. Similarly, the number of FOG symptoms was greater in fallers (*n* = 21) than in nonfallers (*n* = 9) in our study. Furthermore, faller patients had higher NFOG scores, although there was no significant difference between the two groups.

Faller patients showed a greater COP mean distance and wider sway area compared to nonfaller patients (Figure 3). The results indicate that faller patients have inefficient postural balance characteristics, with greater body sway size or more deteriorated postural correction to maintain postural stability against gravity. Especially, a significant group difference was found in the mean ML distance. A significant group difference in sway area was mainly in the ML direction (*r* = 0.84) rather than in the AP direction (*r* = 0.79). In a previous study, an increased ML sway distance was related to an increased risk of falling in normal elderly adults [14]. Our results demonstrated that the amount of lateral sway was also associated with fall risk in PD patients, as well as in normal elderly adults. Similar results were also found in a gait study, in which PD showed greater postural sway in the ML direction [15].

The mean velocity of the faller patients was greater than that of the nonfaller patients in both the AP and ML directions (Figure 2). This indicates that faller patients have faster sway speed, which is related to the amount of regulatory activity, that is, faller patients may require significantly greater activity to achieve postural stability. PD patients showed faster COP velocity compared to healthy elderly adults in both the AP and ML directions [16]. Furthermore, the COP velocity statistically distinguished patients with mild PD from those with moderate PD [16]. Thus, the sway speed component might be helpful in identifying potential fallers in PD patients as well as in distinguishing PD severity.

The peak power is an approximation of the fundamental oscillations in the COP time series and its intensity. In this study, peak power was significantly greater in faller patients than in nonfaller patients (Figure 4). This tendency was especially prominent in the AP direction. The results indicate that faller patients have a greater intensity of the main oscillation component, mainly in the AP direction during static standing. In a previous study, PD patients exhibited greater peak power than healthy elderly adults in both the AP and ML directions [16]. However, our results demonstrated that fall risk was more associated with peak power in the AP direction. In contrast, no significant differences were observed in other frequency variables. In spectral analysis, only AP peak power might be an important indicator.

Many studies have investigated postural balance in PD patients by comparing healthy elderly adults [6, 7]. We focused on a comparison between faller and nonfaller patients and demonstrated that faller patients had different static balance strategies compared to nonfaller patients. The results suggest that ML sway size, AP peak power, and the sway speed obtained from a static standing posture position could be important indicators for identifying potential faller patients among PD patients. Moreover, we demonstrated that these indicators were correlated with clinical balance assessment score (*r* = −0.366∼−0.464, *p* < 0.01).

Physical activity level was associated with postural balance in patients with PD [17]. Furthermore, regular physical activity and exercise habits were significantly associated with slower deterioration of postural stability [18]. It has been reported that higher physical activity level was correlated with more severity of UPDRS III (*r* = 0.63, *p* < 0.01) [19]. In the UPDRS III total score, no significant difference was observed between faller and nonfaller in the present study (*p* = 0.22). This result indicates that the subject with similar physical activity level may have been recruited. Nevertheless, the present study found some quantitative postural balance variables that differentiate faller from nonfaller. However, our study still has limitation that the physical activity level was not directly measured. To consider more accurate physical activity level, acceleration should be also measured during activity of daily living using inertial measurement unit (IMU) sensors as in a further study.

## 4. Conclusions

In conclusion, the results of this study demonstrated the feasibility of identifying potential fallers among PD patients. PD fallers had greater lateral sway size, faster sway speed, and greater AP peak power compared to nonfaller patients. These findings could aid clinicians in identifying potential fallers among PD patients.

## Figures and Tables

**Table 1:** Demographic in each group of subjects.

Subject characteristics	Faller (*n* = 32, men = 14) Mean (SD)	Nonfaller (*n* = 32, men = 14) Mean (SD)	Significance (*p* value)
Age (years)	68.25 (8.44)	67.91 (9.36)	0.88
Height (cm)	159.13 (8.29)	159.31 (7.91)	0.93
Weight (kg)	57.91 (8.81)	60.03 (8.73)	0.35
Disease duration (years)	4.48 (3.62)	2.68 (2.57)	**0.04**
Education (years)	8.48 (4.47)	7.72 (3.38)	0.47
HY stage	2.35 (0.70)	1.95 (0.59)	**0.02**
UPDRS III total score	23.09 (9.87)	19.81 (11.01)	0.22
FOG symptom number	9	21	—
NFOGQ score	17.67 (5.65)	13.78 (10.13)	0.19
Fear of falling score	28.03 (11.55)	10.89 (12.77)	**P** **<** **0.001**
Tinetti_balance	8.88 (3.87)	12.26 (3.48)	**P** **<** **0.001**
K_FAB	12.00 (5.26)	12.93 (3.71)	0.43
PHQ_9	8.86 (5.06)	5.90 (4.37)	**0.02**
STAI	41.50 (11.26)	37.30 (10.29)	0.14

Note: HY stage, Hoehn and Yahr stage; UPDRS, unified Parkinson's disease rating scale; NFOGQ, new freezing of gait questionnaire; K_FAB, frontal assessment battery; PHQ_9, patient health questionnaire-9; STAI, state-trait anxiety inventory; SD, standard deviation. Bold values: *P* < 0.05.

**Table 2:** Comparison of postural balance variables between faller and nonfaller patients.

COP outcome measures	Directions	Faller (*n* = 32, men = 14) Mean (SD)	Nonfaller (*n* = 32, men = 14) Mean (SD)	Significance (*p* value)
Mean velocity (mm/s)	Overall	17.41 (9.45)	11.67 (6.12)	**0.01**
	AP	12.32 (5.94)	8.87 (5.40)	**0.02**
	ML	9.58 (6.95)	5.75 (2.66)	**0.01**

Mean distance (mm)	Overall	7.50 (3.98)	5.67 (1.92)	**0.02**
	AP	4.96 (2.39)	4.07 (1.49)	0.08
	ML	4.54 (2.93)	3.04 (1.45)	**0.01**

Mean frequency (Hz)	Overall	0.40 (0.17)	0.35 (0.16)	0.26
	AP	0.48 (0.19)	0.43 (0.26)	0.31
	ML	0.41 (0.22)	0.37 (0.11)	0.33

Peak frequency (Hz)	Overall	0.40 (0.09)	0.37 (0.07)	0.19
	AP	0.37 (0.10)	0.35 (0.07)	0.30
	ML	0.36 (0.11)	0.34 (0.07)	0.29

Peak power (mm^2^/Hz)	Overall	1031.86 (1130.21)	497.42 (347.01)	**0.01**
	AP	1583.28 (1163.87)	747.42 (351.51)	**P** **<** **0.001**
	ML	1907.72 (2322.22)	843.40 (1376.53)	0.30

Sway area		823.23 (1235.08)	337.20 (192.06)	**0.04**

Note: COP, center of pressure; SD, standard deviation; AP, anterio–posterior; ML, medio–lateral. Bold values: *P* < 0.05.

**Table 3:** Correlation coefficients between COP outcome measures and clinical scores.

COP outcome measures	Directions	UPDRS III	Fear of falling score	Tinetti_balance score
Mean velocity (mm/s)	Overall	0.081	**0.330** ^*∗*^	**−0.427** ^*∗∗*^
	AP	0.143	**0.350** ^*∗∗*^	**−0.420** ^*∗∗*^
	ML	0.012	**0.305** ^*∗*^	**−0.409** ^*∗∗*^

Mean distance (mm)	Overall	0.084	0.231	**−0.366** ^*∗∗*^
	AP	0.118	0.169	−0.236
	ML	0.009	**0.264** ^*∗*^	**−0.464** ^*∗∗*^

Mean frequency (Hz)	Overall	0.074	0.232	−0.135
	AP	0.096	**0.273** ^*∗*^	**−0.281** ^*∗*^
	ML	−0.012	0.053	0.094

Peak frequency (Hz)	Overall	−0.180	0.182	−0.091
	AP	0.151	0.075	0.034
	ML	−0.013	−0.027	0.230

Peak power (mm^2^/Hz)	Overall	0.123	**0.336** ^*∗∗*^	**−0.453** ^*∗∗*^
	AP	0.116	**0.383** ^*∗∗*^	**−0.396** ^*∗∗*^
	ML	−0.006	**0.326** ^*∗*^	**−0.397** ^*∗∗*^

Sway area		0.021	**0.283** ^*∗*^	**−0.428** ^*∗∗*^

Note: COP, center of pressure; SD, standard deviation; AP, anterio–posterior; ML, medio–lateral; ^*∗*^, *p* < 0.05; ^*∗∗*^, *p* < 0.01. Bold values: *P* < 0.05.

## Data Availability

The authors did not receive approval from the IRB to share the data publicly. Hence, the data cannot be shared.
